# Characteristics and Procedures Among Adults Discharged to Hospice After Gastrointestinal Tract Surgery in California

**DOI:** 10.1001/jamanetworkopen.2022.20379

**Published:** 2022-07-06

**Authors:** Anya L. Greenberg, Joseph A. Lin, Alexis Colley, Emily Finlayson, Tasce Bongiovanni, Elizabeth C. Wick

**Affiliations:** 1 Department of Surgery, University of California, San Francisco

## Abstract

**Importance:**

Hospice care is associated with improved quality of life and goal-concordant care. Limited data suggest that provision of hospice services after surgery is suboptimal; however, literature in this domain is in its nascency, leaving gaps in our understanding of patients who enroll in hospice after surgery.

**Objective:**

To characterize the transition to hospice after gastrointestinal tract surgery and identify areas that warrant further attention and intervention.

**Design, Setting, and Participants:**

This retrospective cohort study included patients discharged to hospice after a surgical hospitalization for a digestive disorder in California-licensed hospitals between January 1, 2015, and December 31, 2019. Data were analyzed from August 1 to November 30, 2021.

**Exposures:**

Patient age, race and ethnicity, principal language, payer, and Distressed Community Index (DCI).

**Main Outcomes and Measures:**

Admission type and most common diagnoses and procedures for surgical hospitalizations that resulted in discharge to hospice, annual hospitalization trend for 3 years preceding hospice enrollment, and most common diagnoses for patients who were readmitted after hospice enrollment were summarized. Age, race and ethnicity, principal language, payer, and DCI were compared between patients who were readmitted after hospice enrollment and those who were not.

**Results:**

Of 2688 patients with surgical hospitalizations resulting in discharge to hospice (mean [SD] age, 73.2 [14.7] years; 1459 women [54.3%]), 2389 (88.9%) had urgent or emergent discharges. The most common diagnoses were cancer (primary and metastatic; 1541 [57.3%]) and bowel obstruction (563 [20.9%]). The most common procedures were bowel resection, fecal diversion, inferior vena cava filter, gastric bypass, and paracentesis. In the 3 years preceding hospice enrollment, this cohort had a mean (SD) of 2.21 (2.77) hospitalizations per patient (1537 of 5953 surgical [25.8%]). Of these, 3594 of 5953 total (60.4%) and 840 of 1537 surgical (54.7%) hospitalizations were within 1 year of hospice enrollment. Three hundred and sixty-eight patients (13.7%) were readmitted after hospice enrollment, with infection being the most common readmission diagnosis. Readmitted patients were more likely to be younger (mean [SD] age, 69.7 [16.4] vs 73.8 [14.3] years; *P* < .001), to speak a principal language other than English (62 of 368 [16.8%] vs 292 of 2320 [12.6%]; *P* = .02), to be insured through Medicaid (70 of 368 [19.0%] vs 223 of 2320 [9.6%]; *P* < .001), and to be from a community with higher DCI (198 of 360 [55.0%] vs 1117 of 2269 [49.2%]; *P* = .04) and were less likely to be White (195 of 368 [53.0%] vs 1479 of 2320 [63.8%]; *P* < .001).

**Conclusions and Relevance:**

These findings suggest multiple opportunities for advance care planning in this surgical cohort, with a particular focus on emergent care. Further study is needed to understand the reasons for rehospitalization after hospice discharge and identify ways to improve communication and decision-making support for patients who choose to enroll in hospice care. Given the frequent antecedent interactions with the health care system among this population, longitudinal and tailored approaches may be beneficial to promote equitable end-of-life care; however, further research is needed to clarify barriers and understand differing patient needs.

## Key Points

**Question:**

What are the characteristics of patients who enroll in hospice after undergoing gastrointestinal tract surgery, and what areas of hospice transition warrant further attention?

**Findings:**

In this cohort study of 2688 patients discharged to hospice, most gastrointestinal tract procedures performed immediately before hospice enrollment were for urgent or emergent complications of cancer, with considerable inpatient burden during the 3 preceding years. Patients readmitted after hospice enrollment were more likely to be underserved.

**Meaning:**

These findings suggest multiple opportunities for advance care planning, with a particular focus on emergent care as well as a need for tailored approaches for equitable end-of-life care.

## Teaser

This cohort study characterizes patients who transition to hospice after gastrointestinal tract surgery in acute care hospitals, quantifies the associated use of resources, and identifies areas of hospice transition that warrant further attention and intervention.

## Introduction

Hospice is a component of palliative care intended to optimize quality of life at the end of life using multidisciplinary, team-based care in patients’ preferred settings.^1,2^ In the US, hospice is implemented as an insurance benefit for terminally ill patients whose prognosis for survival is 6 months or less. The hospice insurance benefit pays local agencies to provide comprehensive, symptom-directed care at home or in facilities and bereavement support for loved ones while typically limiting hospital admissions, procedures, and chemotherapy.^3^ Use of hospice has grown during the last decade and is associated with better quality of life and goal-concordant care.^4^

In recognition of these well-established benefits, the American College of Surgeons has endorsed the use of palliative and hospice care for appropriate patients undergoing surgery.^5^ However, emerging data suggest that provision of these services among surgical specialties is inconsistent. For example, timely use of hospice services has been shown to be notably low among patients who died after pancreatic resection.^6^ Moreover, patients receiving surgical care at the Veterans Health Administration were found to be less likely than medical patients in the same system to enroll in hospice before death.^4^ Finally, patients undergoing surgery at 15 hospitals in the Northwest have been found to be less likely than medical patients to receive a palliative care consultation, to have a do-not-resuscitate order in place, and to participate in a discussion about their prognosis, whereas surgical critical care attendings received lower quality-of-dying ratings from nurses and patient families than their medical attending counterparts.^7^ Simultaneously, disparities in access to hospice services have been identified among patients undergoing surgery.^8,9,10^

These emerging data elucidate opportunities to improve end-of-life care for patients undergoing surgery, potentially both in the moment and anticipatory. However, the literature in this domain is in its nascency, and gaps in our understanding of patients undergoing surgery who enroll in hospice remain.^9^ In particular, how patients within different surgical specialties use hospice services, which particular patients benefit from these services, and what resources are used after hospice enrollment are unknown. To begin to address these gaps, we aimed to characterize patients who transition to hospice after gastrointestinal tract surgery in acute care hospitals using a statewide database, quantify the associated use of resources, and identify areas of hospice transition that warrant further attention and intervention.

## Methods

In this retrospective cohort study, we analyzed data from patients discharged to hospice after a surgical hospitalization for a digestive disorder between January 1, 2015, and December 31, 2019. This study was approved by the University of California, San Francisco Institutional Review Board. Informed consent was waived because this study was based on deidentified data from a statewide database. The Strengthening the Reporting of Observational Studies in Epidemiology (STROBE) reporting guideline for cohort studies was followed.

### Data Sources

We used patient discharge data from the California Office of Statewide Health Planning and Development^11^ to identify patients for our analysis. The patient discharge data consist of deidentified patient-level data for each inpatient discharge from a California-licensed hospital, thus reflecting all inpatient discharges from licensed general acute care hospitals in California. Data elements include select patient demographic characteristics (eg, race and ethnicity, payer, 5-digit zip code), facility information, hospitalization characteristics (eg, discharge disposition), and billing data (ie, Major Diagnostic Category [MDC]; *International Classification of Diseases *[*ICD*] diagnosis and procedure; and Medicare severity–diagnosis related group). Given our objective to characterize the transition to hospice and the previously reported racial and ethnic disparities in end-of-life care, race and ethnicity data from the California Office of Statewide Health Planning and Development data set were used in our analysis.

We used the Distressed Community Index (DCI)^12^ as a measure of socioeconomic status. The DCI was developed by the Economic Innovation Group based on the US Census Bureau’s American Community Survey. The DCI is based on a combination of 7 socioeconomic metrics, combined into a holistic measure of a community at the zip code level. The final measure is normalized into a final score ranging from 0 (most prosperous) to 100 (most distressed). Scores may be used continuously or grouped into 5 tiers ranging from prosperous to comfortable, midtier, at risk, and distressed. To simplify analysis, we grouped DCI tiers into 2 categories: prosperous or comfortable vs midtier, at risk, or distressed. This grouping was made pre hoc in recognition of the substantial financial burden faced by middle-class US residents in purchasing health care coverage^13^; alternative categorization was performed as a sensitivity analysis.

### Study Population

We included all adult patients with an MDC-06 category of diseases and disorders of the digestive system, a categorization of surgical based on Medicare severity–diagnosis related group, and discharge disposition of hospice (home or facility) between 2015 and 2019. No exclusions were made based on *ICD* diagnosis code. We excluded a 3-hospital system with outlier readmission rates among patients in hospice suggestive of systematic miscoding. For patients who met our inclusion criteria, associated DCI (0-100) and tier were assigned based on zip code. Using the patient discharge data record linkage number, which is a unique patient identifier, we identified all hospitalizations for each patient during the 3 years preceding hospice enrollment and all hospitalizations after hospice enrollment.

### Variables of Interest and Data Analysis

Data were analyzed from August 1 to November 30, 2021. Descriptive statistics were derived to characterize patients who met our inclusion criteria. Demographics, admission type, and the most common diagnoses and procedures were summarized for surgical hospitalizations that resulted in discharge to hospice. The annual hospitalization trend for the 3 years preceding hospice enrollment was summarized for all hospitalizations and those with a surgical Medicare severity–diagnosis related group only.

The most common diagnoses were summarized for those patients who were readmitted after hospice enrollment. Age, race and ethnicity, language, payer, and distress score were compared between patients who were readmitted after hospice enrollment and those who were not. Two-sided *t* tests and χ^2^ tests were used as appropriate. Hypothesis tests were 2 sided, and the significance threshold was set to *P* < .05. Statistical analyses were performed with Stata/MP, version 16 (StataCorp LLC).

## Results

Our study included 2688 patients (mean [SD] age, 73.2 [14.7] years; 1459 women [54.3%] and 1229 men [45.7%]) who were discharged to hospice after a surgical hospitalization for a digestive disorder between 2015 and 2019 in California. Among these, 5 patients (0.2%) were American Indian or Alaska Native, 217 (8.1%) were Asian or Pacific Islander, 182 (6.8%) were Black or African American, 520 (19.3%) were Hispanic, and 1674 (62.3%) were White. Three hundred fifty-four patients (13.2%) spoke a principal language other than English. Patient characteristics are provided in Table 1. Among patients with surgical hospitalizations resulting in discharge to hospice, 2389 (88.9%) had urgent or emergent admissions. The most common diagnoses were cancer (primary and metastatic, 1541 [57.3%]) and bowel obstruction (563 [20.9%]). The most common procedures were bowel resection, fecal diversion, inferior vena cava filter, gastric bypass, and paracentesis.

**Table 1.** Characteristics of the 2688 Patients Included in the Study Cohort

Characteristic	Data^a^
Age, mean (SD), y	73.2 (14.7)
Race and ethnicity	
American Indian or Alaska Native	5 (0.2)
Asian or Pacific Islander	217 (8.1)
Black or African American	182 (6.8)
Hispanic	520 (19.3)
White	1674 (62.3)
Other^b^	73 (2.7)
Unknown	17 (0.6)
Race and ethnicity (binary)	
Racial and ethnic minority groups^c^	997 (37.1)
White	1674 (62.3)
Unknown	17 (0.6)
Principal language	
English	2332 (86.7)
Language other than English	354 (13.2)
Unknown	2 (0.1)
Payer	
Medicare	1929 (71.8)
Commercial	435 (16.2)
Medi-Cal (Medicaid)	293 (10.9)
Others	22 (0.8)
Self-pay	9 (0.3)
DCI, mean (SD)^d^	42.1 (24.7)
DCI (binary)^e^	
Prosperous or comfortable	1314 (50.0)
Midtier, at risk, or distressed	1315 (50.0)
Admission type	
Emergent or urgent	2389 (88.9)
Elective or scheduled	297 (11.0)
Unknown	2 (0.1)

Abbreviation: DCI, Distressed Community Index.

^a^ Unless otherwise indicated, data are expressed as No. (%) of patients. Percentages have been rounded and may not total 100.

^b^ Category used by the California Office of Statewide Health Planning and Development data set; no additional information available.

^c^ Includes American Indian or Alaska Native, Asian or Pacific Islander, Black or African American, Hispanic, and other race or ethnicity.

^d^ Ranges from 0 to 100, with higher indices indicating greater levels of distress.

^e^ Fifty-nine records did not include zip codes.

In the 3 years preceding hospice enrollment, this cohort had a mean (SD) of 2.21 (2.77) hospitalizations per patient (n = 5953), 1537 (25.8%) of which were categorized as surgical (Table 2). Of these, 3594 (60.4%) total and 840 (54.7%) surgical hospitalizations were within 1 year of hospice enrollment.

**Table 2.** Hospitalizations in Years Leading Up to Final Discharge to Hospice

Hospitalization	Time before month of hospice discharge, y			
	2-3	1-2	<1	Total
**All**				
Total No.	976	1383	3594	5953
Mean (SD) No. per patient discharged to hospice	0.36 (0.91)	0.52 (1.09)	1.34 (1.82)	2.21 (2.77)
Mean (SD) No. per patient with ≥1 hospitalization during period	1.73 (1.26)	1.84 (1.34)	2.38 (1.84)	3.15 (2.82)
**With surgical MS-DRG only**				
Total No.	283	414	840	1537
Mean (SD) No. per patient discharged to hospice	0.11 (0.35)	0.15 (0.44)	0.31 (0.61)	0.57 (0.89)
Mean (SD) No. per patient with ≥1 hospitalization during period	1.15 (0.41)	1.21 (0.49)	1.27 (0.56)	1.48 (0.84)

Abbreviation: MS-DRG, Medicare severity–diagnosis related group.

Among our study population, 368 of 2688 patients (13.7%) were readmitted to a hospital after hospice enrollment (Table 3). The most common readmission diagnosis was infection, followed by acute kidney injury, bowel obstruction, and exacerbation of psychiatric disorder. Readmitted patients were more likely to be younger (mean [SD] age, 69.7 [16.4] vs 73.8 [14.3] years; *P* < .001), to speak a principal language other than English (62 of 368 [16.8%] vs 292 of 2320 [12.6%]; *P* = .02), to be insured through Medicaid (70 of 368 [19.0%] vs 223 of 2320 [9.6%]; *P* < .001), and to be from a community with higher DCI (198 of 360 [55.0%] vs 1117 of 2269 [49.2%]; *P* = .04) and were less likely to be White (195 of 368 [53.0%] vs 1479 of 2320 [63.8%]; *P* < .001).

**Table 3.** Demographic Characteristics of Patients Readmitted to Acute Care Hospitals After Discharge to Hospice

Characteristic	Patient group^a^			% Readmitted	*P* value
	All	Readmitted to hospital after hospice enrollment			
		No	Yes		
Absolute prevalence	2688	2320	368	13.7	NA
Age, mean (SD), y	73.2 (14.7)	73.8 (14.3)	69.7 (16.4)	NA	<.001
Race and ethnicity					
American Indian or Alaska Native	5 (0.2)	5 (0.2)	0	0	<.001
Asian or Pacific Islander	217 (8.1)	180 (7.8)	37 (10.1)	17.1	
Black or African American	182 (6.8)	138 (5.9)	44 (11.9)	24.2	
Hispanic	520 (19.3)	441 (19.0)	79 (21.5)	15.2	
White	1674 (62.3)	1479 (63.7)	195 (53.0)	11.6	
Other^b^	73 (2.7)	60 (2.6)	13 (3.5)	17.8	
Unknown	17 (0.6)	17 (0.7)	0	0	
Race and ethnicity (binary)					
Racial and ethnic minority groups^c^	997 (37.1)	824 (35.5)	173 (47.0)	17.3	<.001
White	1674 (62.3)	1479 (63.7)	195 (53.0)	11.6	
Unknown	17 (0.6)	17 (0.7)	0	0	
Principal language					
English	2332 (86.8)	2027 (87.4)	305 (82.9)	13.1	.02
Language other than English	354 (13.2)	292 (12.6)	62 (16.8)	17.5	
Unknown	2 (0.1)	1 (0.04)	1 (0.3)	50.0	
Payer					
Medicare	1929 (71.8)	1704 (73.4)	225 (61.1)	11.7	<.001
Commercial	435 (16.2)	367 (15.8)	68 (18.5)	15.6	
Medi-Cal (Medicaid)	293 (10.9)	223 (9.6)	70 (19.0)	23.9	
Others	22 (0.8)	19 (0.8)	3 (0.8)	13.6	
Self-pay	9 (0.3)	7 (0.3)	2 (0.5)	22.2	
DCI, mean (SD)^d^	42.1 (24.7)	41.6 (24.7)	45.0 (24.3)	NA	.02
DCI (binary)^e^					
Prosperous or comfortable	1314 (50.0)	1152 (50.8)	162 (45.0)	12.3	.04
Midtier, at risk, or distressed	1315 (50.0)	1117 (49.2)	198 (55.0)	15.1	

Abbreviations: DCI, Distressed Community Index; NA, not applicable.

^a^ Unless otherwise indicated, data are expressed as No. (%) of patients. Percentages have been rounded and may not total 100.

^b^ Category used by the California Office of Statewide Health Planning and Development data set; no additional information available.

^c^ Includes American Indian or Alaska Native, Asian or Pacific Islander, Black or African American, Hispanic, and other race or ethnicity.

^d^ Ranges from 0 to 100, with higher indices indicating higher levels of distress.

^e^ Fifty-nine records did not include zip codes.

Using prosperous or comfortable vs midtier, at-risk, or distressed DCI groupings resulted in a 50-50 split of the cohort. Patients in the midtier, at-risk, or distressed DCI category had a higher rate of readmission (198 of 1315 [15.1%]) than those in the prosperous or comfortable category (162 of 1314 [12.3%]; *P* = .04). In sensitivity analysis, if midtier DCI was recategorized with the prosperous or comfortable tiers, patients in the category of at-risk or distressed DCI alone represented 726 (27.6%) of the cohort and had a higher rate of readmission (111 [15.3%]) but not significantly higher than that of patients with prosperous, comfortable, or midtier DCI (249 [13.1%]; *P* = .14).

## Discussion

In this retrospective cohort study using a statewide database, we characterized patients who transitioned to hospice after gastrointestinal tract surgery. This high-level exploration offers important insights in the context of a limited evidence base and enables us to begin to identify opportunities to improve end-of-life care for patients undergoing surgery.

Our findings demonstrate the considerable burden of end-of-life care in this surgical cohort, including an escalating number of hospitalizations—25.8% being surgical—during the 3 years before hospice enrollment. Moreover, among our cohort, most gastrointestinal tract procedures immediately before hospice enrollment were for urgent or emergent complications of cancer. This finding is striking given the well-established association between aggressive care and worse patient quality of life at the end of life,^14,15,16^ as well as patient preferences to avoid hospitalizations as they approach the end of life.^17,18,19,20^ Simultaneously, patient misconceptions about the likelihood of cure after surgery are pervasive^21^; patient preferences are infrequently explored during preoperative discussions with their surgeon^22^ despite patient sentiments that structured goals of care conversations may be helpful before high-risk surgery^23^; and communication pitfalls have been shown to lead to nonbeneficial or goal-discordant surgery.^24^

Together, these factors stress the importance of preparatory discussions around goals of care as well as more formal advance care planning for patients undergoing surgery. Our longitudinal findings suggest that there are multiple opportunities for such discussions to take place in the time leading up to the end of life. Limited evidence suggests with whom or in what settings patients would prefer to have conversations about goals of care.^22,24^ Intuitively, these conversations will by necessity be difficult for any clinician to initiate regardless of context. Emergent situations in which goal-concordant discussions are particularly challenging are often predated by opportunities for advance care planning. Complications of cancer, which are seen commonly in this cohort, likely occurred for patients with known cancer diagnoses and exposure to previous care, a notion reinforced by the escalating burden of care found in the present study. Although all clinicians must obtain consent from patients based on risks and benefits of their recommended treatment course, these conversations may also serve as opportunities to derive patients’ goals and wishes for future care, which can be documented as advance care planning. Clear documentation and broad accessibility of this information are critical for informed clinical decision-making across multiple settings.^24^ These strategies may ease the uncertainty of emergency surgical situations and may help to better align care with patient goals, particularly when clinicians providing emergency surgery are meeting a patient new to them but with well-documented advance care planning. Although cultural, operational, and educational factors have been raised as barriers to provision of palliative care for patients undergoing surgery,^24,25,26^ specific barriers to advance care planning, goals-of-care discussions, and information sharing need to be better understood.

Further, our findings reiterate and build on the long-standing disparities around end-of-life care seen elsewhere. Differences in access to hospice care by race and ethnicity, social vulnerability, and insurance status have been established in the literature.^2,8,9,10,27,28,29^ Patients in our cohort readmitted after hospice enrollment were less likely to be White, more likely to have limited English proficiency, and more likely to be underinsured, a finding consistent with prior studies.^30,31,32,33^ These disparities are likely multifactorial, arising from demographic biases, clinician biases, language barriers, and cultural considerations.^34,35,36^ In fact, clinicians have been shown to avoid advance care planning with patients of certain racial and ethnic groups and those who have limited English proficiency.^37^ Longitudinal and tailored approaches to advance care planning in vulnerable surgical populations are needed to provide equitable, goal-concordant end-of-life care. Effective approaches will likely require multiple levels of intervention. At the clinician level, implementing standard multidisciplinary workflows around advance care planning discussions may serve as bias interrupters and minimize disparities in who receives advance care planning and how it is delivered. At the health system level, ensuring availability and consistent use of interpreters, advance care materials in multiple languages, and tailored decision aids for patients may facilitate decision-making around end-of-life care and increase informed consent.^37,38,39^ At the community level, engagement of community health workers who are familiar with the history, culture, and norms of the patients they serve may help build patient trust in their care team and facilitate culturally competent advance care planning discussions.^40^ Innovative approaches to engaging historically underserved groups in COVID-19 vaccination programs have emphasized that community-level engagement with religious groups, primary care facilities, and trusted community members can be more effective than physician-led efforts.^41,42,43,44^ To understand next steps for patients who undergo gastrointestinal tract surgery and are discharged to hospice but are readmitted to the hospital, deeper review of clinical documentation to evaluate whether goals of care or advance care planning were attempted or not and even interviews with patients and/or their loved ones to understand positive and negative experiences around discussions will be essential. Importantly, our study uniquely identifies patients from communities with higher DCI as more likely to be readmitted to an acute care hospital after enrollment in hospice. This finding reinforces the role of social determinants of health in disparities in the use of health care services,^45^ suggesting that health care interventions should be complemented by a deep understanding of social context. In this analysis, the midtier DCI was grouped with the at-risk and distressed tiers to create a binary category separate from the prosperous and comfortable tiers. This grouping was chosen pre hoc to reflect the financial burden of health care faced by patients with midtier DCI,^13^ and it incidentally provided a 50-50 split of the cohort. In sensitivity analysis, if midtier DCI was recategorized with the prosperous and comfortable tiers, patients in the category of at-risk and distressed DCI alone represent only 27.6% of the cohort and have a higher rate of readmission but not significantly higher than patients with prosperous, comfortable, and midtier DCI. Nonetheless, the pre hoc grouping of midtier with at-risk and distressed DCI likely better reflects the economic realities of patients undergoing care near the end of life.

### Limitations

Our findings should be interpreted within the context of several limitations. First, because patients who enrolled in hospice after hospital discharge were not included in our study, we very likely underestimated the overall population of patients receiving hospice care proximately after gastrointestinal tract surgery. In addition, we could not identify patients who may have benefitted from but did not enroll in hospice. Second, the data set used did not include information on whether an advance care plan was in place, limiting our ability to understand the role of goal concordance in the care patients received near the end of life. Additionally, there are limitations to using the DCI as a measure of economic status—namely, it is based on the patient’s 5-digit zip code, which is associated with postal delivery and therefore may not be a homogenous community. More granular measures of socioeconomic status exist, such as the Area Deprivation Index (which uses a 9-digit zip code for score assignment).^46^ However, the California Office of Statewide Health Planning and Development data set is limited to the 5-digit zip code, thus limiting our ability to use more granular indices. Nonetheless, the DCI has been shown to have clinical and economic relevance in other settings.^47,48,49^ Despite these limitations, this statewide, multicenter study is a starting point for further exploration of surgical care at the end of life. In particular, future studies should seek to understand not only barriers to advance care planning and goal-concordant discussions but also patient perspectives of when, how, and with whom they would prefer to have them.

## Conclusions

Our findings in this cohort study reveal multiple opportunities for advance care planning among patients who ultimately enroll in hospice after gastrointestinal tract surgery, including escalating hospitalizations and surgical care in the years leading up to hospice. Most gastrointestinal tract procedures immediately before hospice enrollment were for urgent or emergent complications of cancer. Increased rehospitalization after hospice enrollment was seen in underserved patients. Longitudinal and tailored approaches are needed to provide equitable end-of-life care. Further research is needed to clarify barriers and understand differing patient needs.
